# Bone shape mediates the relationship between sex and incident knee osteoarthritis

**DOI:** 10.1186/s12891-018-2251-z

**Published:** 2018-09-12

**Authors:** Barton L. Wise, Jingbo Niu, Yuqing Zhang, Felix Liu, Joyce Pang, John A. Lynch, Nancy E. Lane

**Affiliations:** 1 Department of Internal Medicine, University of California, Davis School of Medicine, Sacramento, CA USA; 2 Department of Orthopaedic Surgery, University of California, Davis School of Medicine, Sacramento, CA USA; 3 Boston University School of Medicine, Boston, MA USA; 4 Department of Epidemiology and Biostatistics, University of California, San Francisco, San Francisco, CA USA; 5 University of New Mexico School of Medicine, Albuquerque, NM USA; 6 Center for Musculoskeletal Health, Departments of Orthopaedic Surgery and Internal Medicine, University of California, Davis School of Medicine, 4625 2nd Avenue, Suite 2002, Sacramento, CA 95817 USA

**Keywords:** Knee, Osteoarthritis, Bone shape, Sex, Statistical shape modeling, Radiography

## Abstract

**Background:**

Knee bone shape differs between men and women and the incidence of knee osteoarthritis (OA) is higher in women than in men. Therefore, the purpose of the present study was to determine whether the observed difference in the incidence of knee radiographic OA (ROA) between men and women is mediated by bone shape.

**Methods:**

We randomly sampled 304 knees from the OAI with incident ROA (i.e., development of Kellgren/Lawrence grade ≥ 2 by month 48) and 304 knees without incident ROA. We characterized distal femur and proximal tibia shape on baseline radiographs using Statistical Shape Modeling. If a specific bone shape was associated with the risk of incident ROA, marginal structural models were generated to assess the mediation effect of that bone shape on the relation of sex and risk of incident knee ROA adjusting for baseline covariates.

**Results:**

Case and control participants were similar by age, sex and race, but case knees were from higher body mass index (BMI) participants (29.4 vs. 27.0; *p* < 0.001). Women had 49% increased odds of incident knee ROA compared with men (adjusted odds ratio (OR) = 1.49, 95% Confidence Interval (C.I.): 1.04, 2.12). There was an inconsistent mediation effect for tibial mode 2 between sex and incident knee ROA, with an indirect effect OR of 0.96 (95% C.I.: 0.91–1.00) and a direct effect OR of 1.56 (95% C.I.: 1.08–2.27), suggesting a protective effect for this mode. Similar findings were also observed for the mediation effect of tibia mode 10 and femur mode 4. These shape modes primarily involved differences in the angular relation of the heads to the shafts of the femur and tibia.

**Conclusions:**

Distal femur and proximal tibia bone shapes partially and inconsistently mediated the relationship between sex and incident knee OA. Women had a higher risk of incident ROA, and specific bone shapes modestly protected them from even higher risk of ROA. The clinical significance of these findings warrant further investigation.

## Background

Osteoarthritis (OA) is the most common form of arthritis, with at least 27 million US adults diagnosed with OA in 2008 [1]. The radiographic appearance of the joint degeneration of knee OA is characterized by a loss of the joint space or loss of articular cartilage in the medial compartment and/or the lateral compartment of the joint. The majority of knee OA is characterized by medial compartment joint space loss, and the radiographic observation of lost joint space width has been confirmed by magnetic resonance imaging that demonstrates loss of cartilage.

Women have a higher prevalence of OA, different patterns of OA in the knee, and an increased risk of total knee arthroplasty due to OA compared with men [2–4] and the explanation for these differences by sex are incompletely explained. Women also have smaller cartilage volume in the knee and this appears to be independent of two known risk factors for knee OA, bone size and body mass [5–7]. A number of factors have been identified that increase the risk of tibio-femoral radiographic OA including obesity, age, female sex, number of childbirths, and African American race, several of which are sex related [8, 9]. Body mass index (BMI) is related to severity of OA in varus alignment but not in valgus alignment [10] and is thus dependent on specifics of malalignment in the knee, which differ by sex. Thus, despite a significant body of research in the epidemiology of knee OA as noted above, comprehensive understanding of the factors that account for the difference in prevalence of knee OA by sex remain unclear.

Our research group and others have reported that knee bone shape differs between men and women without knee OA [7, 11, 12]. However, recently investigators have used Statistical Shape Modeling (SSM) in both two and three dimensions and determined that specific shapes are associated with incident knee OA [13, 14]. Based on these new findings, the aims of this study were to determine whether knee bone shape is associated with risk of knee OA, and to what extent the difference in the incidence of knee radiographic OA (ROA) between men and women is mediated by bone shape.

## Methods

### Study Subjects

Subjects were drawn from the NIH funded cohort the Osteoarthritis Initiative (OAI) which enrolled 4796 participants at baseline who had knee OA or were at high risk of the condition and were aged 45–79, in 4 clinical centers and with a coordinating Center at University of California, San Francisco (more information is available online at https://oai.epi-ucsf.org/datarelease/). Approval for the overall OAI project was given by the institutional review boards at each OAI center, and this project (373289–1) was determined at the IRB at University of California, Davis to be “not human subjects research as defined by Department of Health and Human Services”.

Subjects for the current study were eligible if they had no rheumatoid arthritis, osteonecrosis, or amputation and still had the patella present. Knees were excluded that had been replaced at baseline. In order to be included, knees had to have radiographs available at baseline, 12 month, 24 month, 36 month and 48 month visits. Included knees could not have radiographic knee OA (ROA) at baseline, defined as Kellgren/Lawrence (KL) grade < 2. Cases were knees with incident ROA, defined as KL grade ≥ 2 by the 48 month visit, randomly selected from the universe of such knees within the OAI. Control knees had KL grade < 2 at the 48 month visit, and were frequency matched by age and clinic site to the case knees [11, 15].

### Assessment of bone shape

Bilateral weight-bearing fixed flexion posterior-anterior radiographs were obtained using a plexiglass fixed-frame positioning device at the baseline, 12-month, 24-month, 36-month, and 48-month visits for all subjects. The methods for the SSM methods applied in this study have been previously described [11, 16]. Prior to the shape modeling, all radiographs were reviewed for image quality and sufficient anatomical coverage in the film, and films that had bone edges that extended beyond the border of the radiograph or where poor penetration prevented identification of the edge of the knee were excluded. One reader (JP) outlined the distal end of the femur and proximal tibia using a standardized semi-automatic algorithm on digitized baseline AP radiographs for all knees. Separate shapes were defined for the femur and tibia. SSM were derived for the femur (with 41 points) and the tibia (40 points). Composite femoral and tibial shapes from only the participants analyzed in this study were compiled to generate reference models then used for measuring modes of variation of shape from these references. Mean shape and modes (variations of bone shape) sufficient to explain 95% of total shape variance in this population were derived using principal components analysis; each mode of variation was independent of the other modes of variation. We checked for correlation between modes and found very weak correlation, indicating that the modes were independent of each other. Correlation between modes ranged from − 0.0172 to 0.0218, or the absolute value of correlation between modes ranged from 0.000001–0.0218. We recorded mode scores as the number of standard deviations of that particular mode that the individual knee was away from the mean value for the bone shape mode (constrained to a maximum of ±3 standard deviations in any one mode), and we refer to that as the “standardized score of bone shape”.

### Reliability

Intra-rater reliability was evaluated by repeating measurements in 342 randomly selected subjects and observing the point placement within 2 mm and within 3 mm of the prior knee shape points. The reader was blinded to reliability status and read the repeated radiographs with 5 months of time in-between readings. Intra-rater reliability for the distal femur and proximal tibia were 96.8% and 92.3%, respectively, for point placement within 2 mm and 98.8% and 96.9% for point placement within 3 mm. These results closely parallel reliability results reported in the literature for this type of SSM assessment [17].

### Assessment of incident radiographic knee OA

Knee X-rays were read for Kellgren/Lawrence (KL) grade (0–4) by two experienced readers [15]. The OAI knee radiograph reading data version 0.8 was used in the analysis. Knees were scored for all five visit X-rays concurrently. Baseline visit X-rays were known to readers, but the order of follow-up X-rays was blinded. Readers were blinded to existing clinical or radiological data. Disagreements were adjudicated by an expert panel. Cross-sectional KL grade scores had a kappa of 0.7.

### Assessment of covariates

Information on age, race, clinic site, history of knee injury, and history of knee surgery were collected by questionnaire at the baseline visit of the OAI study. Body mass index (BMI) was calculated using height and weight measurements taken at the baseline visit and applying the appropriate equation to obtain units of kg/m^2^ [18].

### Statistical analysis

First the relation of sex to the risk of incident radiographic knee OA was evaluated using logistic regression model adjusting for age, race, clinic site, history of knee injury and of knee surgery, and BMI. Next, the association of sex with measurement of each bone shape was determined using the linear regression model. Lastly, the relation of each bone shape measurement to the risk of incident radiographic knee OA was evaluated using a logistic regression model adjusting for age, race, clinic site, history of knee injury and of knee surgery, and BMI. Finally, the total effect of sex on risk of incident radiographic knee OA was partitioned into indirect effect (i.e., the effect of sex on incident OA via a specific bone shape; an effect mediated via the effect of sex on bone shape) and direct effect (not mediated through bone shape) using marginal structural models (MSM) [19]. MSM was conducted under a counterfactual framework. In the MSM, sex was an exposure variable, bone shape measurement was the mediator, status of knee OA was an outcome variable, and age, race, clinic site, history of knee injury, history of knee surgery and BMI were covariates. Results are reported as odds ratios (OR) per standard deviation increase in the standardized score of bone shape. SAS v9.2 (SAS, Inc., Cary, North Carolina) was used to complete statistical analyses.

## Results

**Fig. 1:**
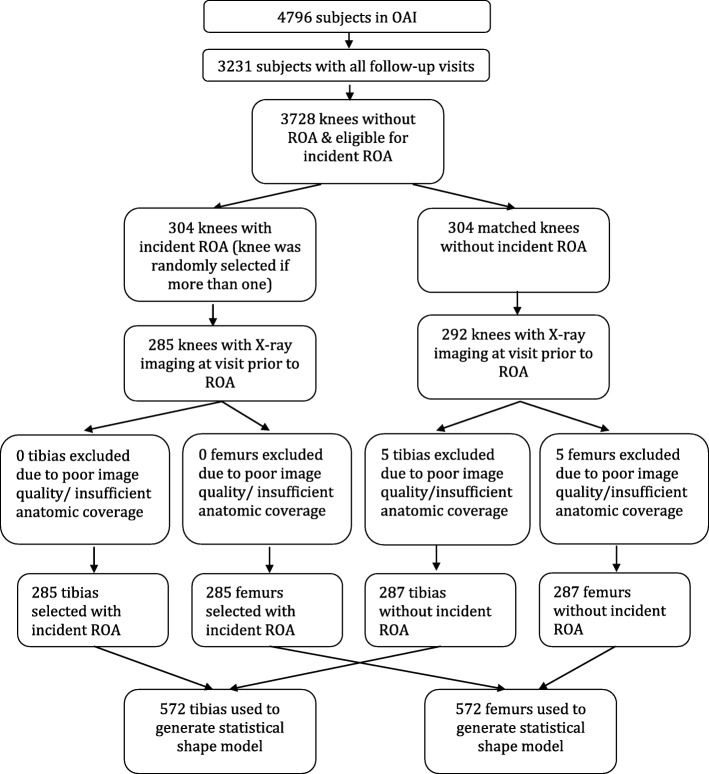
Flowchart for subject inclusion/exclusion

This study included 304 cases of incident knee OA and the same number of controls without radiographic knee OA (see Fig. 1). The percentage of white and mean age in cases were similar to those in controls; however, the proportion of women in cases (65.1%) was slightly higher than that in controls (59.9), and average BMI was greater in cases than that in controls (see Tables 1 and 2). Fifty-eight persons contributed two knees in the study, 21 with cases in both knees, 26 with controls in both knees, and the others with one knee as case and the other knee as control.

**Table 1:** Participant characteristics

Characteristics	Case knees (*N* = 304)	Control knees (*N* = 304)	*p*-value
Baseline age, mean (SD)	60.2 (8.6)	60.2 (8.5)	0.798
Baseline BMI, mean (SD)	29.4 (4.9)	27.0 (4.7)	<.0001
Sex			0.178
Men	106 (34.9%)	123 (40.5%)	
Women	198 (65.1%)	181 (59.5%)	
Race			0.639
Whites or Caucasian	241 (79.3%)	247 (81.3%)	
Black or African American	53 (17.4%)	52 (17.1%)	
Asian	6 (2.0%)	2 (0.7%)	
Other	4 (1.3%)	3 (1.0%)	
Education			0.984
High school or less	33 (11.1%)	36 (11.9%)	
College	140 (47.1%)	138 (45.7%)	
Above college	124 (41.8%)	128 (42.4%)	
Baseline KL grade			
1	162 (53.3%)	45 (14.8%)	<.0001

**Table 2:** Tibia and femur bone shape characteristics

Bone shape mode	Case knees mean (SD)	Control knees mean (SD)
tibia, mode 1	0.004 (0.955)	−0.077 (0.986)
tibia, mode 2	0.073 (1.018)	−0.032 (0.972)
tibia, mode 3	0.048 (1.009)	0.047 (1.007)
tibia, mode 4	−0.034 (0.972)	− 0.017 (0.966)
tibia, mode 5	0.094 (0.980)	−0.058 (0.970)
tibia, mode 6	−0.042 (1.036)	0.015 (0.9765)
tibia, mode 7	−0.005 (1.021)	− 0.042 (0.983)
tibia, mode 8	−0.036 (0.965)	0.043 (1.022)
tibia, mode 9	0.070 (0.978)	−0.069 (1.055)
tibia, mode 10	−0.049 (1.024)	0.067 (1.024)
tibia, mode 11	0.050 (1.019)	− 0.007 (0.987)
tibia, mode 12	−0.057 (0.994)	−0.010 (1.056)
tibia, mode 13	−0.051 (0.944)	0.076 (1.017)
femur, mode 1	0.057 (1.031)	−0.033 (1.006)
femur, mode 2	−0.047 (1.027)	0.054 (0.917)
femur, mode 3	−0.057 (1.033)	− 0.095 (0.999)
femur, mode 4	−0.064 (0.993)	0.088 (0.956)
femur, mode 5	−0.067 (1.043)	−0.009 (0.932)
femur, mode 6	0.048 (1.028)	−0.021 (0.975)
femur, mode 7	0.025 (1.022)	−0.013 (0.994)
femur, mode 8	−0.014 (0.983)	−0.023 (0.983)
femur, mode 9	−0.004 (0.987)	0.080 (0.964)
femur, mode 10	−0.094 (1.001)	0.060 (0.968)
femur, mode 11	−0.058 (1.027)	0.020 (0.975)
femur, mode 12	−0.035 (1.020)	0.017 (0.940)
femur, mode 13	0.001 (1.027)	0.036 (0.960)

OAI subjects as a whole with K/L 0–1 at baseline were 60.3 years old (SD = 9.2) with BMI 27.8 kg/m^2^ (SD = 4.5). 56.2% of them were women, 84.1% were Whites or Caucasian, and 86.3% had college or above college education. The cases/controls in our study were thus slightly more likely to be women and slightly less likely to be Whites or Caucasian. These differences might be due to the fact that they were selected from subjects who came back for the 48-month visit.

**Fig. 2:**
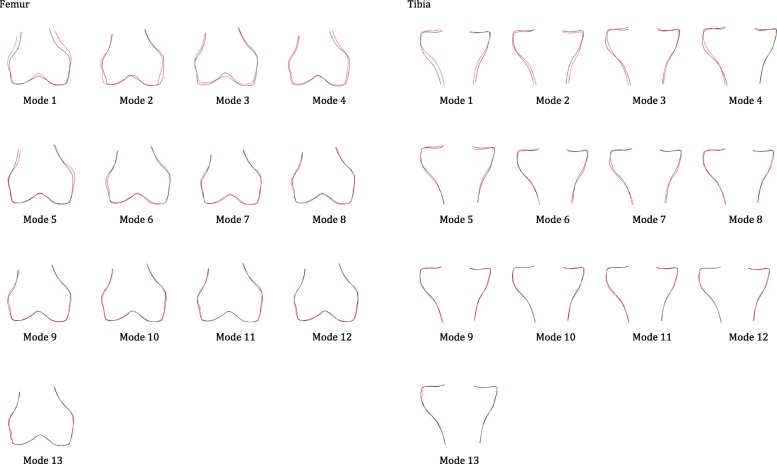
Illustrations of all modes for distal femur and proximal tibia. Bone shapes are shown with + 2 standard deviations (in red outlines) and − 2 standard deviations (in black outlines) for each of the 13 tibia and femur shape modes

Compared with men, women had a higher risk of developing incident knee OA (adjusted OR = 1.49, 95% C.I.: 1.04, 2.12). On average, males who developed incident disease did so 2.08 years after baseline. On average, females who developed incident disease did so 2.11 years after baseline. Of 13 modes of bone shape of tibia and 13 modes of the femur examined (see Fig. 2), statistically significant differences were found between men and women among control knees in modes 2, 3, 8, 10 and 11 in the tibia and modes 1, 4, 5, 8, 12 and 13 in the femur (See Table 3).

**Table 3:** Bone shape mode and sex, incident knee ROA

Mode	Association of mode and incident knee ROA		Mode among knees without incident ROA, by gender	
	OR (95% C.I.)*	*p*-value	Men Mean (SD)	Women Mean (SD)
tibia, mode 1	1.12(0.97,1.29)	0.130	−0.06 (0.93)	−0.11 (1.02)
tibia, mode 2	1.18(1.03,1.37)	0.021	0.20 (1.00)	−0.23 (0.94)**
tibia, mode 3	0.99(0.87,1.13)	0.868	−0.25 (1.05)	0.22 (0.92)**
tibia, mode 4	0.95(0.83,1.09)	0.496	0.001 (0.93)	−0.03 (1.00)
tibia, mode 5	1.12(0.97,1.28)	0.117	0.02 (0.94)	−0.13 (0.97)
tibia, mode 6	0.94(0.82,1.08)	0.400	−0.07 (1.06)	0.05 (0.91)
tibia, mode 7	1.06(0.92,1.22)	0.445	−0.17 (1.05)	0.03 (0.93)
tibia, mode 8	0.94(0.82,1.08)	0.387	0.30 (0.89)	−0.13 (0.07)**
tibia, mode 9	1.15(1.00,1.32)	0.042	−0.06 (1.11)	−0.09 (1.00)
tibia, mode 10	0.79(0.67,0.93)	0.006	−0.21 (0.98)	0.25 (0.98)**
tibia, mode 11	1.04(0.90,1.19)	0.598	0.27 (1.03)	−0.20 (0.91)**
tibia, mode 12	0.86(0.75,0.99)	0.031	−0.004 (1.08)	0.06 (1.04)
tibia, mode 13	0.94(0.82,1.08)	0.381	0.05 (1.01)	0.09 (1.02)
femur, mode 1	1.05(0.90,1.21)	0.552	−0.54 (0.92)	0.30 (0.91)**
femur, mode 2	1.00(0.88,1.15)	0.943	−0.04 (0.86)	0.10 (0.95)
femur, mode 3	1.11(0.96,1.29)	0.170	−0.13 (1.01)	−0.07 (1.02)
femur, mode 4	0.85(0.73,0.98)	0.025	−0.21 (1.00)	0.30 (0.89)**
femur, mode 5	0.95(0.83,1.09)	0.468	0.16 (0.95)	−0.12 (0.91)**
femur, mode 6	1.11(0.97,1.27)	0.147	−0.15 (1.00)	0.02 (0.95)
femur, mode 7	1.01(0.88,1.15)	0.912	0.08 (0.98)	−0.08 (1.01)
femur, mode 8	0.93(0.81,1.08)	0.339	−0.21 (1.01)	0.10 (0.95)**
femur, mode 9	1.01(0.88,1.16)	0.899	0.11 (0.97)	0.02 (0.96)
femur, mode 10	0.81(0.70,0.94)	0.005	−0.09 (0.98)	0.21 (0.95)
femur, mode 11	0.93(0.81,1.06)	0.276	0.04 (0.91)	0.01 (1.01)
femur, mode 12	0.95(0.83,1.08)	0.423	−0.12 (0.83)	0.14 (0.98)**
femur, mode 13	0.94(0.81,1.09)	0.380	0.14 (1.01)	−0.08 (0.90)**

Total effect of sex (women vs. men): OR (95% CI)*= 1.49 (1.04, 2.12)

*Adjusting for age, BMI, race, clinic site, knee injury and surgery

**p-value < 0.05

As shown in Table 3, six modes of bone shape were associated with incident radiographic knee OA after adjusting for age, race, clinic site, history of knee injury and knee surgery and BMI: high values of mode 2 and mode 9 at the tibia were associated with an increased risk of incident knee radiographic OA; however, high values of mode 10 and mode 12 at tibia and mode 4 and mode 10 at femur were associated with a lower risk of knee ROA.

The results of mediation analyses are presented in Table 4. Tibial modes 2 and 10 and femoral mode 4 were found to mediate the relation between sex and ncident knee OA. While the direct effect of sex on risk of knee ROA was greater than 1, indicating that women had a higher risk of knee ROA not including the effect of the specific bone shape mode, the indirect effect of sex on risk of knee ROA in each significant mode was smaller than 1, suggesting that effect of being a woman on bone shape modestly protected them from developing incident knee ROA.

**Table 4:** Mediation analysis results, including indirect and direct effect for each mode

Mediator effect of mode on sex and incident knee ROA	Indirect effect		Direct effect	
	OR (95% C.I.)*	*p*-value	OR (95% C.I.)*	*p*-value
tibia, mode 1	1.00(0.99,1.00)	0.322	1.50(1.03,2.16)	0.033
tibia, mode 2	0.96(0.91,1.00)	0.043	1.56(1.08,2.27)	0.019
tibia, mode 3	0.99(0.97,1.02)	0.642	1.49(1.03,2.16)	0.033
tibia, mode 4	1.00(1.00,1.00)	0.724	1.49(1.03,2.15)	0.035
tibia, mode 5	0.99(0.99,1.00)	0.207	1.49(1.03,2.16)	0.034
tibia, mode 6	0.99(0.98,1.01)	0.576	1.50(1.04,2.18)	0.030
tibia, mode 7	1.00(0.99,1.02)	0.560	1.48(1.02,2.13)	0.038
tibia, mode 8	1.01(0.99,1.03)	0.533	1.46(1.01,2.12)	0.042
tibia, mode 9	0.99(0.99,1.00)	0.119	1.50(1.04,2.17)	0.032
tibia, mode 10	0.96(0.92,1.00)	0.034	1.53(1.05,2.21)	0.025
tibia, mode 11	0.98(0.94,1.02)	0.246	1.53(1.06,2.22)	0.024
tibia, mode 12	0.99(0.99,1.00)	0.145	1.50(1.04,2.17)	0.032
tibia, mode 13	1.00(1.00,1.00)	0.244	1.49(1.03,2.15)	0.035
femur, mode 1	1.03(0.95,1.12)	0.441	1.44(0.98,2.09)	0.061
femur, mode 2	1.00(0.99,1.01)	0.779	1.47(1.01,2.12)	0.042
femur, mode 3	1.01(0.99,1.03)	0.266	1.45(1.00,2.10)	0.049
femur, mode 4	0.97(0.94,1.00)	0.045	1.50(1.03,2.17)	0.034
femur, mode 5	1.01(0.98,1.04)	0.647	1.47(1.01,2.13)	0.043
femur, mode 6	1.00(1.00,1.00)	0.265	1.46(1.01,2.12)	0.043
femur, mode 7	1.00(0.98,1.01)	0.741	1.47(1.01,2.12)	0.042
femur, mode 8	0.98(0.95,1.02)	0.340	1.50(1.03,2.18)	0.032
femur, mode 9	1.00(1.00,1.00)	0.970	1.46(1.01,2.12)	0.043
femur, mode 10	0.97(0.94,1.00)	0.061	1.50(1.03,2.17)	0.034
femur, mode 11	1.01(1.00,1.01)	0.226	1.46(1.01,2.11)	0.046
femur, mode 12	0.99(0.97,1.01)	0.412	1.47(1.02,2.13)	0.040
femur, mode 13	1.01(0.99,1.02)	0.406	1.46(1.01,2.11)	0.046

Total effect of sex (women vs. men): OR (95% CI)*= 1.49 (1.04, 2.12)

*Adjusting for age, race, clinic site, knee injury and surgery, and BMI

**p-value < 0.05

## Discussion

This study confirmed that bone shape in both the tibia and femur differ by sex and that knee bone shape is associated with incident knee ROA. In addition, we made a novel observation that some distal femur and proximal tibia shapes appear to protect women from developing knee OA.

In this study, women had a 49% increased odds of incident ROA compared with men. Our results are in the same range as other reports of sex differences in incident ROA. Felson et al. reported that women had an 80% increased odds of incident ROA in the Framingham group over approximately 9 years, while Muraki et al. reported women had a 58–60% increased odds (depending on K/L grade) of incident ROA over 3.3 years in a Japanese cohort [20, 21]. These study results provide face validity for the current study.

We previously found that femoral mode 4, which mediated the relation of sex with incident knee OA in the current study, was also found to have a borderline significance for difference by sex among persons without OA [11]. Similarly, tibial mode 2 was found to be a mediator for incident knee OA in the current study and represents the same type of shape difference as tibial mode 3 in the prior study of persons without knee ROA, and was found to be significantly different between women and men there. Tibial mode 10 in the present study is similar to tibial mode 10 in the prior study, which was borderline for significant difference by sex. All of these modes that confirm prior related study findings of difference by sex support the validity of our current findings.

The most interesting finding of the current study is that all 3 identified mediating bone shapes exerted a protective effect between sex and incident OA, although the effect size is relatively modest in each case and this size is small enough that there remains some inferential uncertainty as to their meaning. This was an unanticipated finding as bone shape in normal knees is primarily determined by genetics, including chromosomal sex determination [6, 22–26], but the genetic determination of a factor such as bone shape is present prior to exposure to deleterious factors that cause OA, such as pregnancies [9], hormonal effects [27], and joint injuries [28]. Given that these risk factors for OA are common and have existed throughout human evolution, it is likely that natural selection and the evolutionary process would result over time in the appearance of genetic factors (such as bone shape) that could mitigate or protect against risks for OA.

It is not possible to say definitively what elements of the shape difference explain the finding of a protective effect for women without further work. Nonetheless, we did observe that femoral mode 4 primarily involves differences in the relative angle of the condyles to the shaft, with a concomitant alteration of the relative elevation of the articular surface of the medial and lateral condyles. Tibial modes 2 and 10 also appear to involve alterations in the relation of the shaft to the head, again with coincident inverse differences in the relative elevation of the medial versus lateral tibial plateau. With the exception of tibial mode 5, none of the other modes display these types of inverse differences in articular surface elevations. These observations lead us to hypothesize that with activity, knee shape causes force to be offloaded in a compartment-specific manner that may produce the protective effect.

Several prior studies have reported differences in knee bone shape between women and men using a variety of radiographic and measurement approaches [6, 7, 29–31], and each of these studies has reported that men have larger femoral condyle width than women as one of their primary findings. This basic shape difference corresponds with femoral mode 1 in the current study, which was not found to mediate the relationship between sex and incident OA. Other studies have also reported 33–42% greater cartilage volume in men than in women [32], similar to the larger size condyles, but there has been controversy over whether baseline cartilage volume is associated with incident OA [33, 34], and this is complicated by the fact that women may lose cartilage with age more rapidly than men [35]. Thus, the relationship between sex and bone/cartilage size and incident OA are unclear and complex, and the fact that we found no mediating effect for femoral mode 1 suggests that simple condylar size differences are not responsible for the relationship between sex and incident OA. The current study’s report of differences in the relation of the head to the shaft of both femur and tibia as being a mediating factor has not been reported before and identifies a new and potentially fruitful avenue for investigation.

Other joints also display complex relationships between bone shape and OA. In the hip joint, it has long been known that shape abnormalities are associated with radiographic OA [36, 37], and more recently SSM techniques have been employed to establish shapes that are associated with OA [38, 39], but in these studies there has been no examination of differences by sex, let alone shape-related mediation of the association of sex with hip OA. However, the multiple reports of shapes and shape modes predating incident OA establish the concept that genetic variants in bone shape alter the mechanical milieu in a joint and its predisposition to OA. A prior report of differences in the shape of the proximal femur found specific shape modes in the hip were associated with compartment-specific knee osteoarthritis, but that there was no interaction by sex [16]; because shape differences in the hip might affect the knee through alterations of femur shaft biomechanics, the current finding of differences in head orientation to shaft in the femur mediating sex-OA associations is of even greater interest, and suggest the possibility that protective knee bone shapes may have arisen in opposition to mechanical forces that develop at a distance in the bones.

We did not perform sensitivity analyses in the current study. Confounding on the association between sex and knee ROA, sex and bone shape, bone shape and knee ROA all can bias the direct and indirect effect estimates. This makes sensitivity analyses in mediation analysis complex. To make things simple, we calculated E-value for the total, direct and indirect effect based on confounding on the association between sex and knee ROA. E-value is a recently developed measure of the minimum strength of association, on the risk ratio scale, that an unmeasured confounder would need to have with both the treatment and the outcome to fully explain away a specific treatment–outcome association, conditional on the measured covariates [40]. The total effect of sex on incident knee OA was 1.49, and the E-value was 2.34, i.e., the total effect could be explained away by an unmeasured confounder that was associated with both the sex and incident knee OA by a risk ratio of 2.34 fold each or larger. The direct and indirect effect of sex on incident knee OA, for example through tibia mode 2, were 1.56 and 0.96, and the corresponding E-values were 2.49 and 1.25, respectively. However, we do not know of any genetic factor(s) with an RR ≥ 1.25 [41]; thus it is unlikely there is a confounding factor of this size or larger.

The current study has several strengths. We used the OAI cohort, in which radiograph acquisition and reading are standardized and reliable, and in which clinical and demographic characteristics are reliably collected at each time point. Furthermore, the OAI is diverse and representative of populations of both whites and African Americans and thus this study can be considered to be generalizable to persons at risk of knee OA in United States. Lastly, the internal inter- and intra-rater reliability numbers for the SSM for the current study are very good.

The study also has a few limitations. Positioning of study subjects for radiographs could influence the SSM findings which could lead to a misclassification bias; this is despite the extensive efforts made to standardize positioning, beam angle and other elements of radiograph acquisition. The SSM process itself includes a component that is operator dependent which may introduce human error with the potential for unknown effects on derived bone shapes. Final knee alignment data for the entire cohort (including most of the selected knees in this study) was not available at the time of this analysis, so adjustment for this was not possible. Finally, there may have been early OA in the knees chosen even at baseline which was not radiographically observable, but which still might have biased our findings.

## Conclusions

In summary, femoral and tibial knee shapes differ by sex and are associated with incident knee ROA. The shapes of the distal femur and proximal tibia partially and inconsistently mediate the relationship between sex and incident knee OA. Although women had increased risk of incident ROA, their bone shape modestly protects them from having even higher risk.

## Abbreviations

95% C.I.: 95% confidence interval
BMI: Body mass index
KL: Kellgren/Lawrence
MSM: Marginal structural model
OA: Osteoarthritis
OAI: Osteoarthritis Initiative
OR: Odds ratio
ROA: Radiographic osteoarthritis
SSM: Statistical Shape Modeling
